# Guillain-barré syndrome following brainstem infarction: a case report and pathophysiological hypothesis

**DOI:** 10.3389/fimmu.2025.1610219

**Published:** 2025-09-03

**Authors:** Shusheng Jiao, Miaomiao Li, Jianxia Zhi, Zengyang Yu, Xiaofang Cheng, Zihua Gong

**Affiliations:** ^1^ Department of Neurology, Bethune International Peace Hospital, Shijiazhuang, Hebei, China; ^2^ Shijiazhuang 10th Cadre Rest Center of Hebei Military Region, Shijiazhuang, Hebei, China

**Keywords:** guillain-barré syndrome (GBS), albuminocytological dissociation, acute ischemic stroke, brainstem, intravenous immunoglobulin (IVIg)

## Abstract

Guillain-Barré syndrome (GBS), the leading global cause of acquired neuromuscular paralysis, is classically defined as an immune-mediated polyradiculoneuropathy triggered by molecular mimicry between microbial antigens and peripheral nerve components. However, emerging clinical observations challenge the traditional paradigm by reporting GBS following noninfectious events. Notably, the plausible link between GBS and acute ischemic stroke remains unclear, despite isolated case reports suggesting a potential association. Here, we report a rare case of rapidly progressive GBS after acute left pontine infarction. A 72-year-old male with hypertension, type 2 diabetes, coronary heart disease, hyperhomocysteinemia, and a history of ischemic stroke presented with 13-hour acute right-leg weakness and dysarthria. No recent infections were reported. Brain MRI confirmed acute left pontine infarction (DWI hyperintensity/ADC hypointensity). Guideline-based stroke therapy (dual antiplatelet agents, high-intensity statin and comprehensive vascular risk factor management) led to near-complete recovery by Day 12. However, from hospital day 13 onward, he experienced acute neurological deterioration characterized by rapidly progressive flaccid quadriplegia (MRC grade 0/5 in all limbs) and generalized areflexia over five days. Cerebrospinal fluid (CSF) analysis revealed albuminocytological dissociation, and GBS (acute motor axonal neuropathy subtype) was confirmed through nerve conduction studies and electromyography. Serum and CSF anti-ganglioside antibody testing was negative. Intravenous immunoglobulin (IVIG; 0.4 g/kg/day for 5 days) combined with rehabilitation resulted in partial recovery (MRC 2/mRS 4 at 30-day follow-up; MRC 3/mRS 4 at 90-day follow-up). Our findings broaden the etiological spectrum of peripheral demyelinating diseases, and meanwhile highlight that GBS may be an under-recognized cause of post-stroke neurological deterioration, necessitating heightened clinical vigilance. Stroke-induced immunodepression may constitute a biologically plausible mechanistic link bridging cerebral ischemia and subsequent GBS development, and deeper investigation into its pathogenesis is warranted to elucidate its role in stroke-induced GBS variants.

## Introduction

Guillain-Barré syndrome (GBS) represents the leading global cause of acquired flaccid paralysis (1) with an annual incidence of 1–2 cases per 100,000 individuals (2), showing male predominance and age-related susceptibility (3). It is commonly considered as an archetypal postinfectious autoimmune disorder, classically triggered by molecular mimicry between microbial antigens and peripheral nerve components (4). Pathogen-derived epitopes (notably Campylobacter jejuni lipo-oligosaccharides) share structural homology with neural gangliosides, driving cross-reactive antibody production that mediates axonal injury or demyelination (5). Meanwhile, vaccine-induced epitope spreading further expands the etiological spectrum through analogous mimicry mechanisms.

Beyond infectious triggers, GBS is increasingly associated with noninfectious etiologies, including surgical trauma, immunotherapy (e.g., immune checkpoint inhibitors) (6–8), and cerebrovascular events. However, post-stroke GBS remains markedly under-characterized relative to cases associated with surgical interventions or immunotherapy, with significant gaps in both pathophysiological and epidemiological understanding. To date, few hemorrhagic stroke-associated GBS cases have been reported (9), and only a single case triggered by acute ischemic stroke has been described (10). This stark disparity underscores an urgent need for systematic case documentation and rigorous mechanistic studies to clarify possible stroke-GBS causality.

Here, we reported a new case of rapidly progressive GBS following acute pontine infarction. Intriguingly, our case demonstrates remarkable neuroanatomical congruence with the referenced case report, particularly regarding stroke localization within brainstem. This topographical predilection prompts us to propose a novel pathophysiological hypothesis: strategic infarction in brainstem combined with stroke-induced immunodepression may predispose to GBS development.

## Case presentation

A 72-year-old right-handed male presented to our neurology department with acute-onset right lower limb weakness and dysarthria persisting for 13 hours. The patient had a medical history significant for essential hypertension (controlled on amlodipine 5 mg daily), type 2 diabetes mellitus (HbA1c 6.5% on metformin), hyperhomocysteinemia (23.2 μmol/L), and ischemic heart disease, with a prior ischemic stroke occurring 24 months ago. Comprehensive epidemiological investigation revealed no recent (<3 months) history of diarrheal illness, respiratory infections, surgical procedures, or vaccinations.

Upon admission, the patient demonstrated controlled hypertension with stable vital signs. Neurological examination revealed intact cognition and cooperation, right lower limb weakness [proximal/distal Medical Research Council (MRC) grade 4/5], and dysarthria manifested as slurred articulation with mild aspiration during oral intake (Grade 3 on the standardized 3-ml Water Swallowing Test). Motor assessment demonstrated positive pronator drift in the right upper limb, while sensory modalities remained unaffected. Reflex examination showed symmetrical deep tendon responses (2+) with bilateral Chaddock signs and equivocal Babinski responses; remaining physical examination findings were unremarkable. On admission, this patient had a National Institutes of Health Stroke Scale (NIHSS) score of 3 (1 point for right leg motor weakness, 1 point for dysarthria) and a modified Rankin Scale (mRS) score of 2, indicating slight disability with preserved independence in basic activities of daily living.

**Figure 1:**
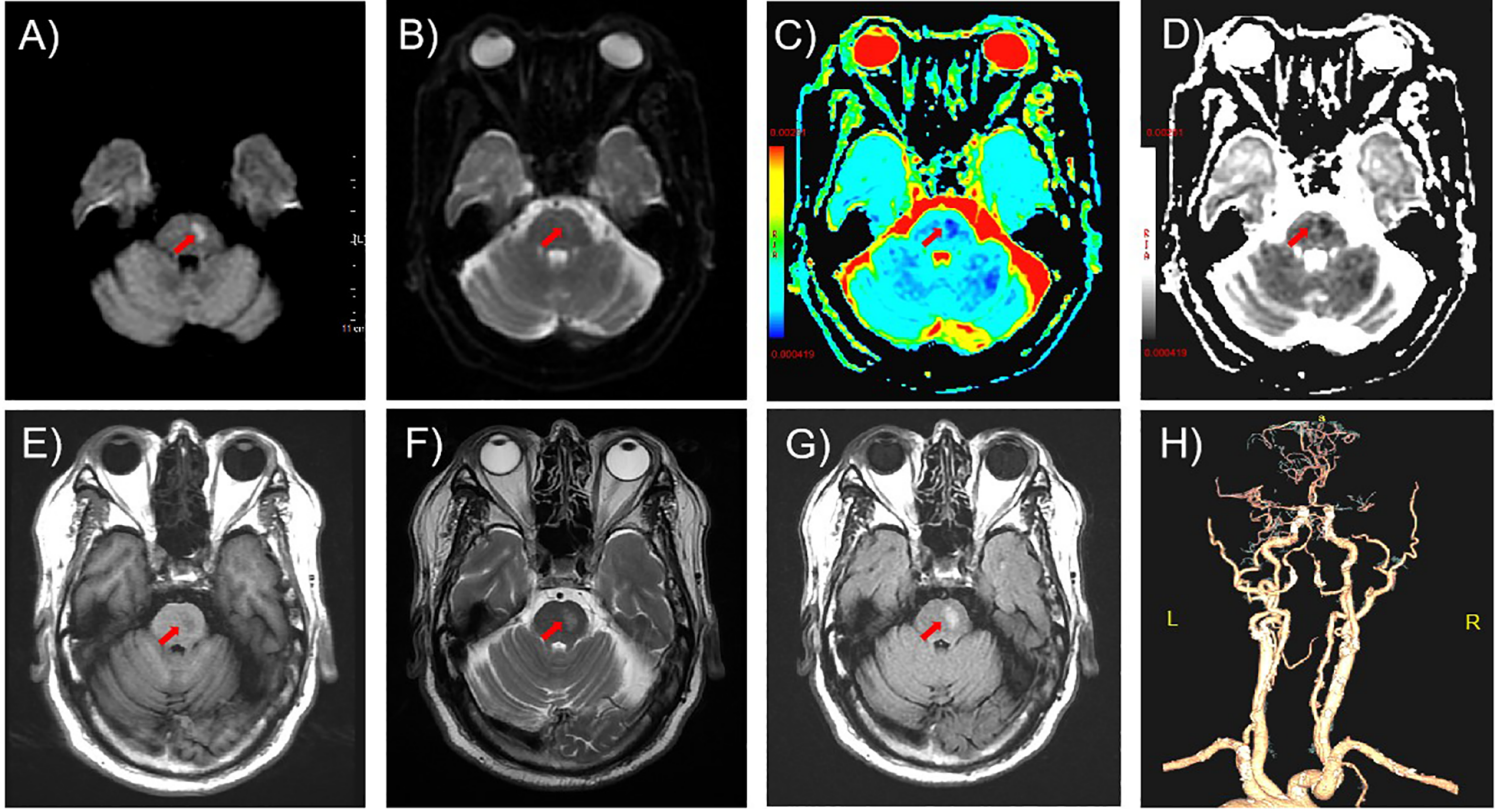
Neuroimaging findings on admission. **(A-D)** Axial diffusion-weighted imaging (DWI) sequences revealed restricted diffusion in the left pontine region (arrows): **(A)** hyperintensity on DWI (b=1000 s/mm²), **(B)** isointensity on b=0 image, **(C, D)** corresponding hypointensity on apparent diffusion coefficient (ADC) mapping. **(E)** T1-weighted imaging demonstrated focal hypointensity (arrow). **(F, G)** Axial T2-weighted and fluid-attenuated inversion recovery (FLAIR) sequences showed confluent hyperintensity (arrows). **(H)** Computed tomography angiography (CTA) revealed subtotal occlusion of the right M1 middle cerebral artery (MCA) segment and multifocal severe stenoses (>70%) involving the basilar artery and bilateral vertebral arteries.

Initial non-contrast brain CT, performed immediately upon arrival in the Neurology outpatient clinic (time: +13.5 hours post-onset), excluded hemorrhage and space-occupying lesions. Subsequent brain MRI [including Diffusion Weighted Imaging (DWI)/Apparent Diffusion Coefficient (ADC)] obtained 30 minutes post-CT revealed an acute pontine infarction (DWI hyperintensity, ADC hypointensity) with corresponding T1/T2 signal alterations (**Figures 1A-G**), accompanied by Fazekas grade 1 white matter changes and mild cortical atrophy. Neurovascular evaluation (CT angiogram, CTA), performed after permission to inpatient ward (time: +2 days post-onset) demonstrated critical cerebrovascular pathology: subtotal occlusion in M1 segment of right middle cerebral artery (MCA) and multiple severe stenosis in both basilar artery and bilateral vertebral arteries (**Figure 1F**).

**Figure 2:**
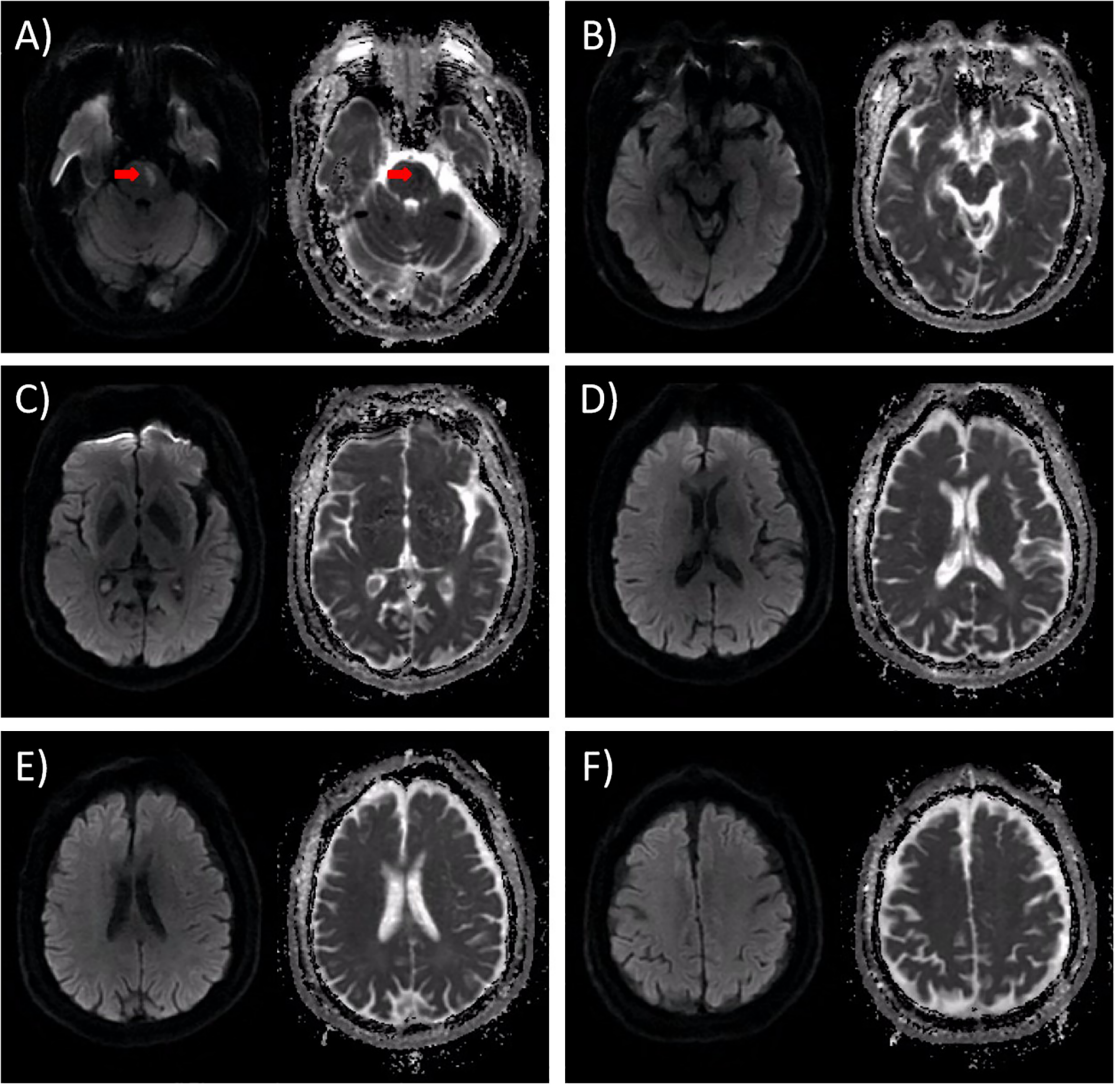
DWI results obtained on the 13th day following admission. The left panel depicts the DWI image, while the right panel shows the corresponding ADC image. **(A)** In the original infarct lesion situated in the left pons, the DWI signal exhibited a subtle reduction, accompanied by a moderate increase in the ADC value. Notably, there was no observable enlargement of the lesion, as indicated by the red arrow. **(B-F)** Upon thorough examination, no new infarct lesions or signs of hemorrhagic transformation were detected. Specifically, **(B)** represents the peduncle level of the axial section; **(C)** corresponds to the basal ganglia level of the axial section; **(D)** denotes the lateral ventricle level of the axial section; E indicates the corona radiata level of the axial section; and **(F)** signifies the centrum semiovale level of the axial section.

The patient was diagnosed with acute left pontine ischemic stroke [Trial of Org 10172 in Acute Stroke Treatment (TOAST) classification: large artery atherosclerosis] based on clinical and neuroimaging findings. The culprit lesion responsible for the acute presentation was severe basilar artery stenosis. The patient did not receive intravenous thrombolysis due to presentation beyond the ≤4.5-hour therapeutic window. Endovascular thrombectomy was offered but declined by the patient and family, leading to initiation of standard medical therapy including antiplatelet agents and aggressive vascular risk factor modification. Given the patient’s NIHSS score ≤3 and severe stenosis of the culprit vessel, we implemented guideline-directed therapy including: dual antiplatelet therapy (DAPT) with aspirin (100 mg/day) and clopidogrel (75 mg/day), initiated within 24 hours of symptom onset and maintained for 21 days per 2023 AHA/ASA recommendations; high-intensity statin therapy (atorvastatin 40 mg nightly); homocysteine-lowering therapy; comprehensive vascular risk factor management targeting LDL <70 mg/dL, BP <130/80 mmHg and HbA1c <7%. Following 12 days of guideline-directed medical therapy, neurological deficits achieved near-complete resolution, quantified by NIHSS 1 (minimal residual dysarthria), mRS 1 and Kubota Water Swallow Test Grade 2. However, on day 13, he developed rapidly progressive flaccid quadriplegia with universal areflexia over 5 days, without autonomic/brainstem involvement. Repeat MRI excluded new ischemic/hemorrhagic lesions (**Figure 2**). Diagnostic workup revealed albuminocytological dissociation (CSF protein 1.17 g/L, WBC 4×10^6^/L) and electrophysiological evidence of motor axonal neuropathy (CMAP amplitudes <20% lower limit of normal (LLN) in all examined nerves except the right peroneal nerve, reduced F-wave persistence) (**Tables 1**, **2**). Serological and CSF anti-ganglioside antibody panels were negative. Confirmed as acute motor axonal neuropathy (AMAN) subtype of Guillain-Barré syndrome, he received Intravenous immunoglobulin (IVIg) (0.4 g/kg/day ×5 days) with partial motor recovery (MRC 2/mRS 4) after 30 days. At the 90-day follow-up, the patient continued to exhibit residual limb weakness, with MRC 3/5 in proximal and distal segments of all four limbs.

**Table 1:** The results of motor nerve conduction studies.

Nerve (site/muscle)	Distal latencies (ms)	CMAP amplitude (mV)	CMAP duration (ms)	Distance (mm)	Conduction velocity (m/s)
Left median nerve					
Wrist - APB	4.0	0.72	7.0		
Elbow-Wrist	8.2	0.18	6.8	230	54.8
Right median nerve					
Wrist - APB	5.3	0.95	7.0		
Elbow-Wrist	10.3	0.44	6.5	230	45.5
Left ulnar nerve					
Wrist-ADM	3.6	0.49	8.1		
Bl.elbow-Wrist	9.5	0.07	2.6	320	54.2
Right ulnar nerve					
Wrist-ADM	3.9	0.73	7.3		
Bl.elbow-Wrist	10.4	0.14	7.0	320	49.6
Left peroneal nerve					
EDB-Ankle	6.4	0.33	5.3		
Fibular head-Ankle	18.4	0.04	6.9	330	27.6
Right peroneal nerve					
EDB-Ankle	6.4	0.55	5.4		
Fibular head-Ankle	19.0	0.22	4.5	330	26.1
Left tibial nerve					
Ankle-Abductor hallucis	8.7	0.13	9.6		
Knee-Ankle	20.5	0.05	2.9	420	35.4
Right tibial nerve					
Ankle-Abductor hallucis	9.2	0.34	5.3		
Knee-Ankle	23.0	0.05	2.6	420	30.4

CMAP, compound muscle action potential; APB, abductor pollicis brevis; ADM, abductor digiti minimi; EDB, extensor digitorum brevis.

**Table 2:** The results of sensory nerve conduction studies.

Nerve	Distal latencies (ms)	SNAP amplitude (uV)	Distance (mm)	Conduction velocity (m/s)
Left median nerve	2.8	25.50	150	53.6
Right median nerve	3.2	23.88	150	47.5
Left ulnar nerve	2.3	23.68	110	48.7
Right ulnar nerve	2.2	22.61	110	50.9
Left sural nerve				No response
Right sural nerve	4.0	6.58	150	37.7
Left superficial peroneal nerve				No response
Right superfical peroneal nerve	2.8	10.13	120	43.2

SNAP, sensory nerve action patentials.

## Discussion

As the leading cause of acute flaccid paralysis globally, GBS encompasses heterogeneous subtypes with distinct clinico-pathological profiles. Expanding the spectrum of noninfectious precipitants is critical for elucidating its immunopathogenesis. Notably, we present a rapidly progressive GBS case following acute ischemic stroke, revealing a previously underrecognized temporal association between cerebrovascular events and post-stroke autoimmune neuropathy. This association suggests novel neurovascular-immune crosstalk mechanisms requiring further investigation.

Stroke-induced immunodepression syndrome (SIDS), a well-documented phenomenon characterized by transient immunosuppression following cerebral ischemia (11), may provide a plausible mechanism for the observed temporal association between stroke and GBS. SIDS typically involves lymphocytopenia, impaired lymphocyte function, and a shift toward anti-inflammatory cytokines (12). This immunosuppressive state could compromise immune surveillance and increase susceptibility to subclinical infections, potentially lowering the threshold for dysregulated immune responses such as GBS in genetically predisposed individuals. Although speculative in this case, the temporal correlation and documented lymphocytopenia (declining from 1.37×10^9^/L on day 1 to 1.27×10^9^/L on day 14) suggest SIDS may contribute to the immune dysregulation implicated in GBS pathogenesis.

Moreover, two additional stroke-related clinical scenarios merit consideration for their potential pathophysiological relevance, as their underlying mechanisms may offer critical insights into understanding ischemic stroke-triggered GBS. Compared to ischemic stroke, hemorrhagic stroke is associated with a higher reported incidence of subsequent GBS based on current literature. While intracerebral hemorrhage triggering GBS has been reported in multiple brain regions, hemorrhage in the basal ganglia predominates as the most frequent site (9). The proposed mechanism involves erythrocyte breakdown releasing ganglioside-mimicking glycoproteins, triggering anti-ganglioside disialo-1a (GD1a) antibody production. Evidence indicates that cardiac surgery elevates GBS risk, with perioperative cerebral hypoperfusion documented in 68% of affected (13). This supports the emerging ‘double-hit’ hypothesis: surgical stress primes systemic immune activation, whereas concurrent stroke disrupts the blood-nerve barrier (BBB), facilitating pathogenic antibody penetration into peripheral nerves.

We noted a recent publication in Frontiers in Immunology titled “Case Report: Guillain-Barré Syndrome Following Acute Ischemic Brainstem Stroke,” (10) which describes a case strikingly similar to our reported patient. This observation underscores that post-stroke GBS is not an isolated phenomenon. Specifically, the striking similarity in the stroke localization (brainstem region) between their case and ours suggests that specific neuroanatomical sites of infarction may predispose to GBS development. The shared involvement of the brainstem across these cases raises the possibility that unique pathophysiological mechanisms—such as neuroinflammatory cascades, molecular mimicry targeting brainstem-peripheral nerve antigens, or autonomic dysregulation—may underlie this association. We now propose hypotheses linking brainstem ischemia to immune-mediated peripheral nerve injury, including: (1) BBB disruption and antigen exposure. Ischemia-reperfusion injury following brainstem infarction compromises BBB integrity, facilitating the leakage of central nervous system (CNS)-derived antigens [e.g., ganglioside monosialo-1(GM1)/GD1a] into systemic circulation (14). Notably, brainstem neurons exhibit enriched expression of these gangliosides, which share structural homology with peripheral nerve axolemmal antigens (9). BBB breakdown enables antigen-presenting cells to recognize these antigens, triggering the production of cross-reactive autoantibodies (e.g., anti-GM1 IgG). (2) Neuroinflammatory cascades and spillover. Post-infarction neuroinflammatory cascades exacerbate GBS pathogenesis through a coordinated interplay of immune mechanisms. Ischemia-induced microglial activation initiates the inflammatory cascade by releasing pro-inflammatory cytokines [interleukin (IL)-1β, IL-6, tumor necrosis factor (TNF)-α], which enhance macrophage infiltration and major histocompatibility complex (MHC)-II-mediated antigen presentation to peripheral T cells (15). Concurrently, chemokines recruit Th1 cells and monocytes to peripheral nerves via the compromised BBB (16), establishing a self-perpetuating inflammatory loop that accelerates demyelination and axonal degeneration. Complementing these processes, CNS antigens leaking through the disrupted BBB activate peripheral B-cell clones via epitope spreading (17), generating cross-reactive antibodies targeting critical nodal and axonal structures such as the nodes of Ranvier. (3) Autonomic instability. Brainstem infarction induces profound autonomic dysregulation that critically modulates neuroinflammatory responses through dual immunomodulatory pathways. Medullary lesions trigger sympathoexcitation by reducing vagal tone and augmenting sympathetic outflow, leading to elevated systemic norepinephrine levels (18). This catecholamine surge enhances Th1 lymphocyte polarization via β2-adrenergic receptor signaling, promoting interferon-gamma (IFN)-γ-mediated demyelination. Concurrently, diminished vagal activity impairs the cholinergic anti-inflammatory pathway, attenuating α7 nicotinic acetylcholine receptor (α7nAChR)-dependent suppression of splenic macrophages. The resultant disinhibition unleashes uncontrolled pro-inflammatory cytokine production (e.g., IL-6, TNF-α), while impaired regulatory T-cell function further exacerbates peripheral nerve autoimmunity (19). These reciprocal mechanisms—sympathetic hyperactivation amplifying effector T-cell responses and parasympathetic failure permitting macrophage-driven inflammation—collectively establish a self-reinforcing cycle that potentiates both central neuroinflammation and peripheral nerve injury (20).

This patient had a 3-year history of type 2 diabetes mellitus managed with long-term metformin therapy (1500 mg daily), with recent glycated hemoglobin (HbA1c) maintained at 6.5%. Comprehensive review of systems revealed no symptoms of limb numbness, pain, or weakness over this period. These findings preclude confirmation of long-standing diabetic peripheral neuropathy, which can also present with limb weakness and absent tendon reflexes. Emerging evidence suggests that diabetes likely served as a synergistic predisposing factor for GBS in this case: Dima et al. demonstrated diabetes-triggered focal GBS manifesting as acute bilateral phrenic neuropathy (21); Bae et al. established that diabetes exacerbates clinical and electrophysiological severity of GBS (22); Peric et al. confirmed diabetes worsens short-term GBS outcomes (23). These evidence also directly elucidates two key features in our patient: rapid progression to tetraplegia within 5 days and protracted recovery (mRS 4 at 3-month follow-up). Therefore, we posit that diabetes mellitus acts as a disease-modifying promoter in GBS, exacerbating immune-mediated nerve injury through hyperglycemia-induced BBB disruption and impaired axonal regeneration, thereby amplifying both progression velocity and recovery duration.

While this patient exhibited hyperhomocysteinemia, a well-established vascular risk factor, its potential association with GBS remains speculative. Current evidence robustly links elevated homocysteine to ischemic stroke mechanisms, but no convincing pathophysiological or epidemiological data support its direct or indirect involvement in GBS pathogenesis.

## Conclusion

We report a rare GBS occurrence following acute ischemic stroke, expanding the etiological spectrum of peripheral demyelinating disorders. Critically, our observations challenge the traditional autoimmune-centric paradigm of GBS, advocating for integrated pathophysiological models encompassing cerebrovascular contributions. Additionally, GBS is frequently overlooked in stroke patients experiencing neurological deterioration. Our findings propose GBS as a potential etiology of post-stroke neurological decline and emphasize the necessity to consider noninfectious triggers (e.g., ischemic stroke) in GBS diagnostic evaluations. This association warrants mechanistic investigations to delineate neurovascular-immune interactions in dual CNS-peripheral nervous system (PNS) pathologies.

## Data Availability

The raw data supporting the conclusions of this article will be made available by the authors, without undue reservation.
